# Visuospatial task-related prefrontal activity is correlated with negative symptoms in schizophrenia

**DOI:** 10.1038/s41598-019-45893-7

**Published:** 2019-07-03

**Authors:** Adrian Curtin, Junfeng Sun, Qiangfeng Zhao, Banu Onaral, Jijun Wang, Shanbao Tong, Hasan Ayaz

**Affiliations:** 1 Drexel University, School of Biomedical Engineering, Science and Health Systems, Philadelphia, PA USA; 2 Shanghai Jiao Tong University, School of Biomedical Engineering, Shanghai, China; 3 Shanghai Mental Health Center, Shanghai Jiao Tong University, School of Medicine, Shanghai, China; 4 University of Pennsylvania, Department of Family and Community Health, Philadelphia, PA USA; 5 Children’s Hospital of Philadelphia, Center for Injury Research and Prevention, Philadelphia, PA USA

**Keywords:** Attention, Schizophrenia

## Abstract

Control of attention is thought to be specifically impaired in schizophrenia due to abnormal function in the prefrontal cortex (PFC). The PFC plays a critical role in the identification of relevant stimuli and the development of appropriate biases for the identified signals, including selection of an appropriate attentional ‘zoom’. We examined how demands associated with changes in attentional requirements in a Sustained Attention Task (SAT) may contribute to differences in functional involvement of the PFC and relation to clinical status. A group of 24 individuals with schizophrenia and 16 healthy controls (N = 40) performed the SAT and a visuospatial condition (vSAT) while activity in the bilateral anterior PFC was monitored using functional Near Infrared Spectroscopy (fNIRS). The results confirm that the right frontopolar region plays a role in control of attention for both patients and healthy controls. However, patients with schizophrenia exhibited a general attentional deficit and inefficient right-medial PFC activation. Additionally, we observed a strong regional association between left Middle Frontal Gyrus (MFG) activity during the vSAT task and the PANSS score driven by the negative symptom subscale. The presence of aberrant activation differences within the left-MFG region may describe a dysregulation of attentional networks linked to the clinical expression of negative and general symptoms.

## Introduction

Schizophrenia is a debilitating brain disorder characterized by positive, negative, and cognitive symptoms that affects approximately 1% of the global population^1^. Attention deficits are among the key cognitive impairments associated with the illness and are a driving factor in quality of life and other functional outcomes^2^. These symptoms typically predate psychosis and may act as a stable indicator of schizophrenia vulnerability^3^ but are largely unresponsive to pharmaceutical intervention with traditional or atypical neuroleptics^4^. Without any appropriate avenue for remediation, attention impairment represents a fundamental cognitive symptom that is unaddressed by currently available clinical therapies^5^. Illuminating the interactions between neural dynamics and attentional deficits may offer insight into neuro-individualized approaches to therapy.

Neuroimaging studies investigating the impairment of attention have found that behavioral deficits in schizophrenia are observed in conjunction with abnormal activation patterns within cortical regions strongly associated with attention such as the dorsolateral prefrontal cortex (dlPFC), anterior prefrontal cortex (aPFC), anterior cingulate cortex (Acc), the insula, and other subcortical regions^6,7^. In particular, dysfunction in the prefrontal cortex (PFC) is commonly observed as diminished activation, termed “hypofrontality”^8^. However, both insufficient activation (decreased) and excessive activation (increased) in the PFC have been reported in patients^9,10^, suggesting that rather than an explicit failure of function, irregular involvement of the PFC may reflect dysregulation in the processes of attention itself. Evidence from cued target tasks and saccadic responses indicate that the bottom-up maintenance of processes is relatively intact within schizophrenia^11^, while in the presence of unpredictable cues and stimuli, the top-down processes are impaired^12^. As a result, it has been proposed that people with schizophrenia may be particularly impaired during the “Control of Attention”^12^, a top-down process through which information is restricted to relevant stimuli and appropriate attentional biases are developed^13^ including selection of an appropriate attentional ‘zoom’^12^. Attentional biases towards task-relevant information are theorized to occur through the coordination of top-down and bottom-up mechanisms according to the biased competition model of selective attention^13^. Researchers have sought to tease out the differences between these affected processes in order to quantify and evaluate the control of attention as a candidate marker for drug development^14^.

Attention can be conceived as a limited cognitive resource which must be allocated appropriately in order to operate efficiently^15^. ‘Control of Attention’ guides this allocation as described in part by the “Spotlight” or “Zoom Lens” theory of attention, which postulates that attention operates on a continuum between two stages. In the first stage, attention is fairly evenly distributed over the central field of a view to allow for parallel processing of an area. Whereas in the second stage, attention becomes focused on a specific area, leading to improved focal awareness at the expense of more distributed stimuli^16^. Thus, there exists a tradeoff between processing efficiency and the size of the area being attended according to the level of ‘zoom’ that is engaged during a task. In schizophrenia, an inefficient ability to define or maintain appropriate attentional distribution may lead to a state of “hyperfocusing”^17^ which may reduce the visual span^18^ or saccadic length^19^, and even contribute to failures in spatial working memory^20^.

A recent Arterial Spin Labeling (ASL) imaging study using the Sustained Attention Task (SAT) identified a role for the right-PFC (BA 9/10) in healthy subjects when task difficulty was modulated by the presence of a distractor^21^ and it was later proposed that dysfunction in this region may be the cause of observed behavioral deficits in patients with schizophrenia^22^. The right-PFC region has previously been ascribed a substantial role in initiation and maintenance of attentional processes^23^ as well as attentional dysfunction associated with fatigue^24^ and distraction^21^. In schizophrenia, studies of attentional processes have noted the specific involvement of the right-PFC as an important regional hub^25^ and observed failures in attention have been as ascribed to a “frontal inefficiency” stemming from diminished connectivity between the executive network guided by the dlPFC and a salience network driven by the anterior insula^26^. Functional studies have also identified this region as an area of interest; whether due to dysfunctional changes in lateralization of activity^27^, failure to modulate with attention demands^28^, or even as an area which is increasingly engaged following cognitive remediation^29^.

We hypothesized that sensitivity to differences between broader attentional distribution and focused attention, as measured by contrasting a visuospatial SAT with the standard SAT, could create a candidate assessment for attentional control deficits in schizophrenia. Furthermore, we hypothesized that activity in the right-PFC would reflect the sensitivity of attentional processes to increased visuospatial demands, and that this sensitivity would be dysregulated in schizophrenia in a way which may provide clinically useful information. To test these hypotheses we sought to monitor task-evoked activities with functional Near Infrared Spectroscopy (fNIRS), a non-invasive neuroimaging technique which measures relative changes in cortical hemoglobin concentrations associated with cognitive activity^30,31^. We used linear mixed effects (LME) models to evaluate the relationship between prefrontal activation, cognitive function, and clinical measures.

## Methods

### Demographic information

Participates in this study consisted of 16 healthy controls (10 female) recruited from the local community and 24 patients (16 female) diagnosed with schizophrenia who were recruited from the Shanghai Mental Health Center in Shanghai, China. Participants were all native Chinese over 18 years of age who voluntarily gave informed and written consent to participate in the study. The protocol in this study was reviewed, approved, and carried out according to the guidelines of the IRB of the Shanghai Mental Health. No significant differences were found between group age, education, or gender. All subjects participated in the HAM-D 24 Depression Scales and HAM-A Anxiety Scales. Patients were assessed according to the PANSS and its subscales. Patients scored significantly higher than controls on HAM Depression (p = 0.001) and Anxiety scores (p < 0.01) respectively. Demographic information is presented in Table 1. Detailed antipsychotic medication at time of testing for participants is listed in Supplementary Table A2.

**Table 1:** Demographic and clinical severity information for participants in study.

		Patients (N = 24)		Controls (N = 16)		Comparison
Demographic Information	Sex	8 Male	16 Female	6 Male	10 Female	NS
		*Mean*	*Std*	*Mean*	*Std*	
	Age (years)	35.63	(13.54)	36.06	(12.29)	NS
	Education (years)	10.83	(3.52)	13.81	(3.82)	NS
Clinical Information	Duration Ill (years)	11.36	(10.90)	—	—	
	PANSS	71.65	(17.92)	—	—	
	Positive	17	(6.32)	—	—	
	Negative	18.46	(6.72)	—	—	
	General	34.92	(10.31)	—	—	
	HAM-D 24	8.92	(9.49)	0.56	(0.89)	p = 0.001
	HAM-A	2.96	(4.16)	0.31	(0.70)	p < 0.1
	Severity (SI)	3.58	(1.10)	—	—	
	Global (GI)	2.92	(1.06)	—	—	
	CPZe dosage	297.2	(177.87)	—	—	

PANSS = Positive and Negative Syndrome Scale.

HAM-D = Hamilton Scale for Depression.

HAM-A = Hamilton Scale for Anxiety.

CPZe = Chlorpromazine equivalent dosage in mg.

Parentheses indicate standard deviation (SD).

### Experimental procedure

**Figure 1:**
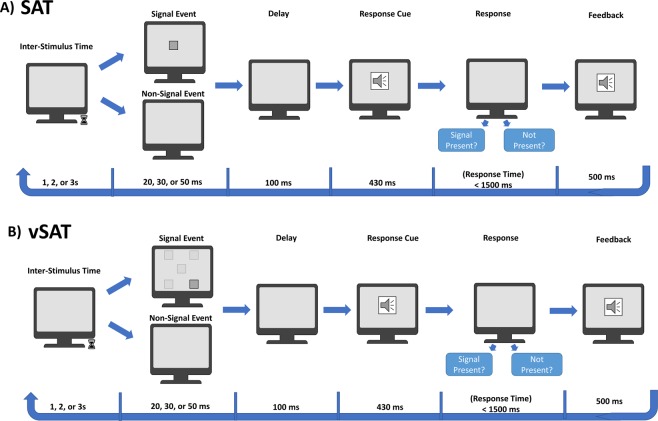
Protocol for implementation of the sustained attention task (SAT) trial (**A**) and the visuospatial SAT (vSAT) trial (**B**) (Based on modified task from Demeter *et al*.^32^).

The SAT was constructed using a custom OpenGL program based on the methodology detailed in Demeter *et al*.^32^. Participants were asked to monitor a silver screen (Lum 170) for a signal consisting of the appearance of a small gray square (Lum 107) in the center of the screen for varying durations of 20, 30 or 50 ms (corresponding to 1, 2, and 3 frames at a typical refresh rate of 60 Hz). Monitoring times before signal/non-signal presentation were varied pseudo randomly at a 1, 2, or 3 s inter-trial interval, and signal frequency was roughly 50% (Fig. 1). 100 ms following the appearance or non-appearance of the signal, the subject was informed by an audio cue and asked to denote whether the event was a signal or non-signal using the left and right mouse click respectively. The subject then was given 1500 ms to classify the event. Correct classifications were immediately given an audio feedback, while incorrect trials receive no feedback. Each task block was preceded by a 30 second resting period and lasted for approximately 120 seconds with approximately 42 stimulus trials in each task block. Following the end of each block, a cumulative performance metric is displayed to the subject. Each subject performed a total of 4 blocks, with two repetitions for each task type (vSAT & SAT).

A modification to the SAT, detailed here as vSAT, was used to introduce broadened attentional focus through the introduction of a visuospatial component. The task in vSAT is similar to the VSL-SAT task described by Demeter *et al*.^22^ with two modifications intended to increase task difficulty by requiring additional attentional resources. Contrast between the silver background and the signal was reduced by using a lighter gray block (Lum 155) to reduce the prominence of the signal. Additionally, signal location was pseudo randomly presented at one of 5 separate locations on the screen (Center, Top-Left, Top-Right, Bottom-Right, and Bottom-Left). An illustration describing the protocol is presented in Fig. 1.

Task performance was assessed using the SAT score, calculated using the methodology defined by Demeter *et al*.^22^. This metric, which had been previously identified as the “Vigilance Index”^33^, was identified as being comparable to other signal-detection measurements, namely d-prime^21^, and uninfluenced by errors of omission. SAT score is defined as

$${\rm{Score}}=\frac{{\rm{Hits}}-{\rm{False}}\,{\rm{Alarms}}}{[{\rm{2}}\,\ast \,({\rm{Hits}}+{\rm{False}}\,{\rm{Alarms}})-{({\rm{Hits}}+{\rm{False}}{\rm{Alarms}})}^{{\rm{2}}}]},$$

where **Hits** mean probability of correct signal/non-signal classification and False Alarms denote probability of incorrect signal/non-signal classification. SAT score ranges from −1 to + 1, with +1 indicating correct classification of all signal/non-signal events and −1 indicating that all responses were false alarms.

Additionally, we calculated the Score Difference as a measure of performance changes associated with Task Type manipulation. Score Difference (*ScoreDiff*_*vSAT*_) was calculated as the difference between Score on the vSAT task and the subject’s mean SAT score. Larger values of *ScoreDiff*_*vSAT*_ are undesirable and would indicate large performance decreases associated with task type variation and increased sensitivity to attentional demand manipulation.

### fNIRS data acquisition and processing

Neuroimaging data was collected for 16 channels at 2 Hz from the anterior PFC (BA 10/46) using the fNIR1100 imager (fNIR Devices, Potomac, MD, USA) using COBI Studio as described in detail at Ayaz *et al*.^34^. The fNIRS sensor was symmetrically centered and in line with Fp1 and Fp2 of the international 10–20 system on the subject’s forehead. The light intensity and detector gain were adjusted during setup to obtain sufficiently high but unsaturated intensity signals according to subject and environmental conditions. Channels were visually inspected for signal validity^34^ and the signal quality of individual task blocks was assessed and automatically rejected using the criteria adapted from Takizawa *et al*.^35^. Surviving blocks were screened for motion artifacts using the SMAR algorithm^36^, and a bandpass filter (0.008–0.25 Hz) was used to reduce high frequency noise associated with respiratory or cardiac function and remove signal drift^37^. Physiological parameters are calculated using the modified Beer-Lambert Law^38^ using the 5 seconds before task initiation as a baseline period. Extracted measures are reported as the fNIRS biomarkers of [HbO], [HbR], and an estimate of relative change in Oxygen availability [Oxy] (calculated as the difference between [HbO] and [HbR])^39^. The mean change from the pre-task baseline in fNIRS biomarkers ([HbO], [HbR], and [Oxy]) was assessed as the outcome of interest for statistical tests. Parametric plots for visualization purposes were registered to a virtual brain according to the methodology described in Ayaz *et al*.^40^. Estimated underlying gyri and Brodmann’s areas for Optodes 1–16 are described in the Supplementary Materials of Liu *et al*.^41^.

### Statistical analysis

Data analysis was conducted using R (ver. 3.2.2) and SPSS (ver. 23.0.0, SPSS Inc.). Descriptive demographic and clinical statistics are presented as mean ± standard deviation or as a percentage of cases. An alpha level of α = 0.05 was used as the significance criteria for each test. Differences between clinical scores and demographic metrics were tested using the Welch’s t-test except for gender distribution which was assessed using the Chi-square test. Correlations for continuous variables are reported using Spearman’s rank correlation coefficients for identified groups as two-tailed test. Correlational analysis for biomarkers was controlled for Family-wise error rate using a channel-wise false discovery rate (FDR)^42^. Comparisons between behavioral results and published results presented in the appendix were made using unpaired t-tests.

Linear mixed-effects (LME) models were constructed to estimate the effects of task types across subject group as well as clinical covariates on observed behavioral metrics and fNIRS biomarkers. LME models offer significant advantages over repeated measures ANOVA in neuroimaging applications as they do not require equal numbers of observations and allow the models to be fit to individual participants^43–46^. Models were analyzed using the maximum likelihood (ML) function and degrees if freedom were approximated using Satterthwaite approximation using the R package “lme4”^47^. Significance values were thresholded for family error rate using an optode-wise false discovery rate^48^.

Models here are reported in a similar style to R syntax. LME models for the dependent behavioral metrics of Response Time and SAT-Score were constructed with Task Type, Clinical Group, and Duration as candidate fixed effects and Subject as a random effect with a slope fit for each Block.

Behavioral models were constructed from the initial models:

$$(\mathrm{SAT}-{\rm{Score}},{\rm{Mean}}\,{\rm{Response}}\,\mathrm{Time})={Group}\,\ast \,Task\,Type\,\ast \,Duration+(Block|Subject)$$

To simplify the interpretation of the behavioral models, terms removed through the examination of the full model permutations and the optimal model based on the Akaike Information Criterion (AIC) was analyzed^49^. Models were with an AIC score of difference greater than 2 were considered to be statistically different^50^.

In order to test the effect of task parameters and clinical status on cerebral hemodynamic biomarkers, [HbO], [Hb], and [Oxy] were assessed as dependent measures in separate LME models. Three models were constructed in order to examine the general response to task performance (1), performance sensitivity within the vSAT (2), and the contribution of clinical status to observed activity (3).

In the first model, the presence of group differences during task performance was estimated using Task Type, Score, and Clinical Group as fixed effects:

$${\rm{Model}}\,{\rm{1}}:{\rm{\Delta }}[X]={Group}\,\ast \,{Task}\,{Type}\,\ast \,{Score}+(1|{Subject})$$

In the second model, the relationship between cerebral hemodynamics during the vSAT and individualized susceptibility to attentional modulation was assessed using Score Difference and Group as fixed effects:

$${\rm{Model}}\,2:{\rm{\Delta }}[{X}]={Group}\,\ast \,{Score}\,{Difference}+(1|{Subject})$$

In the third model, we examined the influence of clinical status using Task-Type and total PANSS score as fixed effects:

$${\rm{Model}}\,3:{\rm{\Delta }}[X]=PANSS\,\ast \,{Task}\,{Type}+(1|{Subject})$$

Selected fNIRS spatial parametric maps are presented using the calculated F-statistics and thresholded using statistical significance for visualization purposes for individual model parameters. Statistical significance of model fixed effects was assessed using the Satterthwaite approximation for degrees of freedom.

## Results

### Behavioral results

#### Task type, stimulus duration, and subject group modulate task performance

**Figure 2:**
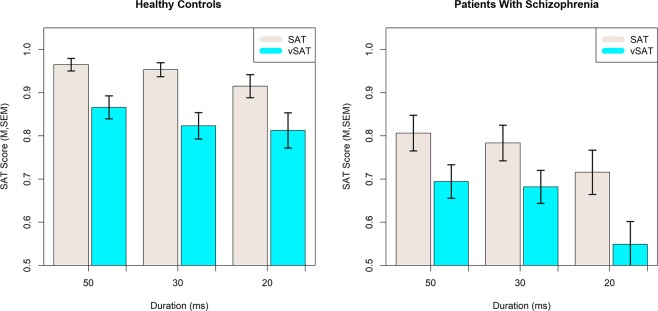
Effects of Task Type and Stimulus Duration on the SAT Score by healthy adult controls and patients with schizophrenia.

We constructed an LME model to assess the influence of subject Group, Task Type, and stimulus Duration on behavioral measures using block number as a random slope and Subject as a random effect. Group was treated as a between-subject factor, while Task-Type and Duration were included as within-subject factors. In the reduced model there was a fixed effect for Group on SAT-score (F(1,37.56) = 9.496, p = 0.0038) with Patients performing at an estimated 16 percent lower than Controls. There was a significant fixed effect for SAT type on SAT-score (F(1,258.08) = 40.093, p < 0.0001) with the more difficult vSAT resulting in an estimated 5 percent reduction in Score. A significant fixed effect was also observed for stimulus duration (F(2,386.97) = 12.142, p < 0.0001) where performance decreased as duration decreased from 50 ms to 20 ms. No interactions between Task-Type, Group, or Duration were significant for Score and these terms were not present in the selected optimal model. The behavioral measures of SAT Score across duration and task type are presented in Fig. 2. Inclusion of block number in the LME model did not provide either a significant fixed effect for block number (F(1,37.09) = 0.079, p > 0.7) or an improved model (ChiSq = 0.837, p > 0.7) suggesting that SAT-Score was not influenced by block number.

#### Response time strongly differs between clinical groups and is modulated by task conditions

**Figure 3:**
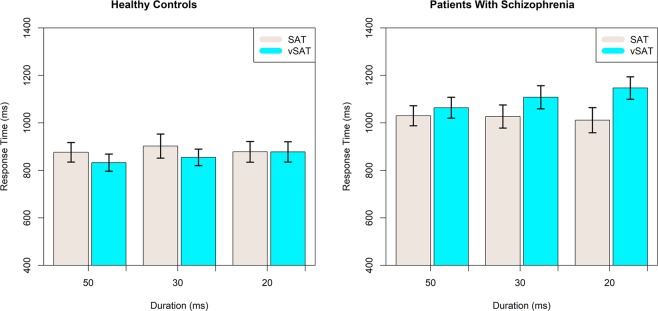
Effects of Stimulus Duration and Task Type on Mean Response Time for healthy controls and patients with schizophrenia.

A significant fixed effect on mean response time was observed for Group (F(1,37.14) = 8.644, p = 0.0056) and a significant interaction for Task Type and stimulus Duration (F(5,395.50) = 10.046, p < 0.0001) with patients having an estimated delay in average response time of 173 ms. Despite very strong significance for the interaction term for Task Type and Duration, the interpretation of the model terms is not straightforward. In general, it is observed that shorter stimuli duration increased response time most clearly in the vSAT task. Effect of stimulus Duration and Task-type for each subject Group on the mean response time are presented in Fig. 3. Model results for Behavioral results including parameter estimates are presented in Table 2.

**Table 2:** LME parameter estimates for SAT-Score and Mean Response Time.

Outcome	Main Effects					Parameter Estimates			
	df_num	df_den	F	p		Linear		Quadratic	
***SAT-Score***									
Intercept				0.0000	***	0.8865	(0.0482)		
Class	1	37.56	9.496	0.0038	**	−0.1919	(0.0623)		
Task-Type	1	258.08	40.093	0.0000	***	−0.0811	(0.0128)		
Duration	2	386.97	12.142	0.0000	***	−0.0645	(0.0137)	−0.0197	(0.0137)
***Mean Response Time***									
Intercept				0.0000	***	763.07	(463.07)		
Class	1	37.14	8.644	0.0056	***	173.77	(59.03)		
Task × Duration	5	395.9	10.0459	0.0000	***				
20 ms × SAT				0.0612	†	−32.59	(17.36)		
30 ms × SAT				0.0380		15.27	(17.36)		
50 ms × SAT				0.0002	***	64.35	(17.36)		
30 ms × vSAT				0.0004	***	56.87	(15.95)		
50 ms × vSAT				0.0151	*	22.87	(15.95)		

Denominator Degrees of freedom calculated with Satterthwaite approximation.

Random effects specified as random intercept and slope for each participant.

^†^p < 0.1 **p < 0.01.

*p < 0.05 ***p < 0.001.

### fNIRS results

#### Right-medial prefrontal role for task performance and interaction with group

**Figure 4:**
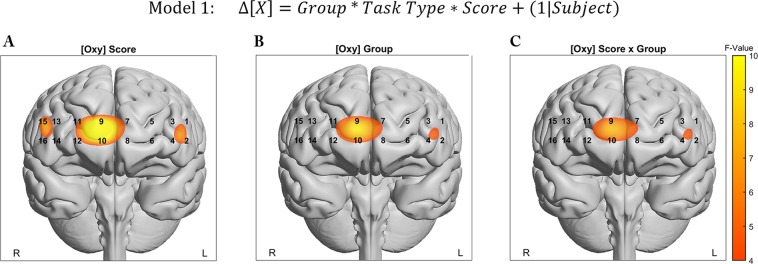
Parametric representation of significant model term overlaid onto anatomical reference images. Values represent mixed model F-Values thresholded. (**A**) Behavioral SAT Score, (**B**) Subject Group, (**C**) Interaction between Score and Group.

In order to ascertain the role of the prefrontal cortex associated with variations in task type, we constructed LME models to test the hypothesis that subject Group, Score, and Task Type were related to mean biomarker changes during task performance. We observed a strong relationship between [Oxy] and the fixed effect of SAT-Score in Optodes 9, and 10 in the right-medial and right-medial-orbital Superior Frontal Gyrus (SFG) respectively. Both optodes showed significant fixed effects for Score following FDR correction (F(1,110.26) = 10.47, q = 0.012, F(1,87.16) = 10.21, q = 0.016). Additionally, Optodes 9 and 10 showed uncorrected significant effects for Group (F = 6.29~6.98, p < 0.009~0.014) and the Score and Group interaction (F = 5.17~5.57, p < 0.02~0.025). The model term for the interaction of Group and Task Type approached significance for Optode 9 (F = 3.62, p = 0.059). The Biomarkers for [HbO] and [HbR] individually did not identify any Optodes that survived FDR thresholding. Parametric Maps are presented for significant model terms in Fig. 4. Model results for Optode 9 are presented in Table 3.

**Table 3:** LME parameter estimates for [Oxy] Optode 9.

Outcome	Main Effects					Parameter Estimates	
	df_num	df_den	F	p		Linear	
***[Oxy] Optode 9***							
Intercept				0.0047	**	1.4006	(0.4860)
Group	1	110.92	6.9817	0.0094	**	−1.3404	(0.5073)
Task-Type	1	109.79	1.9087	0.1699		−1.1614	(0.6826)
Score	1	110.26	10.4671	0.0016	**	−1.5634	(0.5268)
Group × Task	1	109.79	3.6239	0.0597	†	1.35	(0.71)
Group × Score	1	110.26	5.5686	0.0200	*	1.32	(0.56)
Task × Score	1	109.39	1.4607	0.2294		1.16	(0.74)
Group × Task × Score	1	109.39	3.1433	0.0790	†	−1.38	(0.78)

Denominator Degrees of freedom calculated with Satterthwaite approximation.

Random effects specified as random intercept for each participant.

^†^p < 0.1 **p < 0.01.

*p < 0.05 ***p < 0.001.

#### Sensitivity to attentional workload localized to right-medial-SFG

While no significant model terms were observed for Task Type in biomarkers typically associated with activation ([HbO], [Oxy]), we sought to determine whether performance sensitivity to task changes was related to prefrontal cortical activity. To examine this, we defined *Score Difference* as the difference between the mean score obtained during the SAT task minus the subject’s score obtained during the vSAT task. This Score Difference was taken as a measure of a subject’s sensitivity to increased task monitoring difficulty adjusted for baseline attentional performance. A larger Score Difference would imply that the subject experienced more difficulty under the vSAT condition while a smaller score difference would imply that the subject either successfully adapted to task difficulty changes or did not find the task to be more challenging. As a measure of performance on the vSAT task, we analyzed neuroimaging data in the context of the vSAT task periods only.

**Figure 5:**
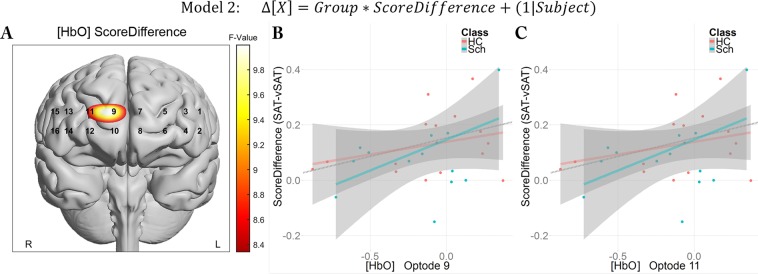
*Score Difference* shows role for performance sensitivity across clinical groups in Optodes 9 and 11. (**A**) Full model parametric maps overlaid onto anatomical reference image. Values represent mixed model F-Values thresholded using FDR. Correlations are presented for both groups in Optode 9 (**B**) and Optode 11 (**C**) (rho = 0.41, p = 0.016~0.024).

In examining the effect of sensitivity to attentional demand on task performance changes, we observed a broad uncorrected significance for *Score Difference* in many of the medial optodes (3,7,9,10,11,12) for the biomarker [HbO], but only Optodes 9 and 11 survived FDR thresholding (F(1,56) = 10.00, q = 0.02; F(1,46) = 8.46, q = 0.044). Score Difference also survived FDR thresholding in Optode 9 for the [Oxy] biomarker (F(1,53.57) = 9.68, q = 0.048). These differences did not appear to differ across subject Group as inferred both by similar regression lines in both groups, and lack of significance in the Group or interaction terms in the linear models. No optodes were identified as significant for changes in the [HbR] biomarker. Visualization of FDR-thresholded significant model terms and pertinent correlations are presented in Fig. 5.

#### vSAT-evoked activity shows relationship to clinical status in Left-MFG

**Figure 6:**
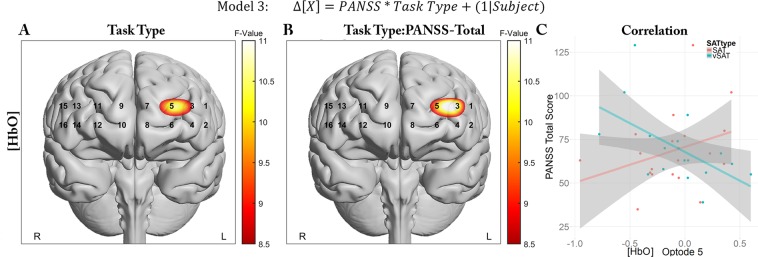
Differing relationship between clinical status and left-Prefrontal activity between tasks types (SAT vs. vSAT). Parametric representation of significant model term overlaid onto anatomical reference images. Values represent mixed model F-Values thresholded with FDR. (**A**) Task Type, (**B**) Task and PANSS interaction, (**C**) Correlations between Optode5 and PANSS total for both tasks.

Biomarker models were constructed to evaluate total PANSS score and the relationship between cortical response to Task Type and clinical status. Following FDR correction, we observed significant fixed effects for Task Type for [HbO] in Optode 3 and 5, near the left Middle Frontal Gyrus (MFG), as well as the interaction between Task Type and PANSS in Optodes 3 and 5 for [HbO] (F(1,54.85) = 8.65, q = 0.038; F(1,52.3) = 10.42, q = 0.017) and Optode 3 for [Oxy] (F(1,71) = 9.44, q = 0.048). No optodes were identified as significant when [HbR] was investigated as a biomarker. Significant model terms and selected correlations are presented in Fig. 6.

**Figure 7:**
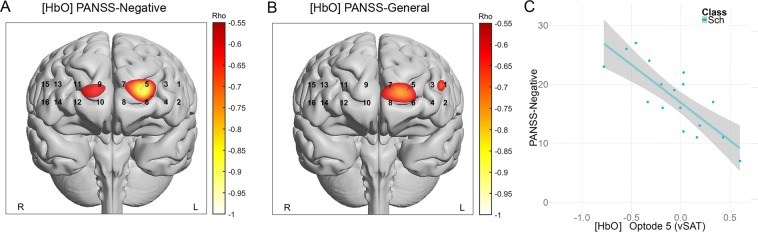
PANSS Subscales exhibited strong correlations with left-Prefrontal activity during vSAT Task Condition. Correlational parametric maps overlaid onto anatomical reference images in panels (A,B). Values represent significant correlations strength as measured by Spearman’s Rank Correlation between [HbO] and the (**A**) PANSS Negative subscale, and (**B**) PANSS-General Subscale. (**C**) Correlations for Optode 5 with PANSS Negative for vSAT task (rho = −0.776, p = 0.0002).

To investigate the basis of these relationships, we explored the correlations of both optodes with PANSS total and the Negative, Positive, and General subscales, and the mean biomarkers for each subject. We observed significant correlations at Optode 5 for [HbO] (rho = −0.58, p = 0.014) during the vSAT task which appeared to be related to a strong negative correlation with the PANSS Negative subscale (rho = −0.77, p = 0.0002) and a weaker correlation with the General subscale (rho = −0.46, p = 0.06). Marginally significant correlations during the SAT condition were observed at Optodes 3 and 5 for [HbO] with the PANSS positive subscale (rho = 0.45~0.484, p = 0.035~0.04) but not for other subscales. Parametric distribution of subscale correlation values and supporting correlations are presented in Fig. 7. Additional correlations for other optodes with behavioral performance, duration of illness, equivalent antipsychotic dosage and other clinical outcome measures are reported in the Supporting Materials (A2).

## Discussion

The sustained attention task (SAT) is a key task in the CNTRICS project which had identified the ‘Control of Attention’ as a principle impairment in schizophrenia and proposed the measurement of this impairment as a key biomarker in drug development^14^. The test consists primarily of a signal/non-signal classification paradigm, following a brief tone and has been validated to modulate with distraction and stimuli length^33^, and has demonstrated performance sensitivity to a variety of drugs including benzodiazepine, acetylcholine receptor ligands, and 192 IgG-saporin^51^. The task has been translated for use in human experimentation with the goal of producing a task which can be relevant at both the animal and human level of neural systems^52^. This cross-species evaluation potentially provides a closer understanding of the relation between neurotransmitter systems and human functional neuroimaging, as well a tool for evaluating the efficacy of procognitive pharmaceuticals under development.

In this study, we noninvasively monitored activity in the bilateral anterior PFC using fNIRS during an implementation of the SAT and a revised version (vSAT) to explore differences in broad vs narrow attention performance in schizophrenia. Results confirm a general attentional deficit in patients with schizophrenia as demonstrated by decreased task performance and increased response times relative to healthy controls. Compared with the healthy controls, patients exhibited reduced performance on both task variants and increased response times on average. We also observed an apparent decrease in task performance with shorter stimulus intervals in both subject groups. Both healthy and patient participants demonstrated relative performance decreases in response to increased task demands and results were not dependent on the specific performance metric used, as both classification accuracy and d-Prime metrics revealed similar behaviors as discussed in the Supporting Materials (A1). Response Times did not appear to be sensitive to task conditions in either group. Behavior performance results as measured by SAT-Score, largely resembled those observed by Demeter *et al*.^22^ in both Healthy and Clinical Groups. This suggested that the present implementation of the SAT task was largely consistent with previous work, although notably, we did not observe a significant Task Condition x Group interaction in the current study. Comparison with previously published results is explored in detail in the Supporting Materials (A3).

Task performance and Group appeared to play significant roles in observed hemodynamic activity evoked in right-medial regions of the PFC (medial Superior Frontal Gyrus). Score and membership in the clinical Group were estimated as a negative modifier to changes in [Oxy], with a stronger estimated negative slope for Score in Healthy Subjects. This finding would point to a relative dysregulation in schizophrenia as a failure to efficiently deactivate the right-medial SFG in a manner which increases Task Performance in an area known to be associated with sustained attention^24^. The anterior PFC, approximating Brodmann’s Area 10, has been frequently associated with both with the control of attention^53^ and more broadly with other higher order executive functions including decision making, working memory, problem-solving, and mentalizing responses^54^.

We observed a role in the right-medial SFG for performance sensitivity as measured by the relative decrease in SAT-score induced by the vSAT condition (Score Difference). We observed that right-PFC activity was differentially modified in response to increased demands with larger relative performance decreases associated with an increase in right-medial SFG activation. One possible interpretation for this is this additional activation is related to the increased difficulty of maintaining a broader area of attention or a wider attentional ‘zoom’. In a continuous arterial spin labeling (ASL) study conducted by Demeter *et*.*al*.^21^, the authors observed right-MFG involvement in the sensitivity of performance to a distraction condition using a variation of the SAT in healthy subjects. Therefore, this increased activity could point to an increase in required attentional effort in response to task demands, despite an inability to maintain performance relative to initial task conditions. The presence of these similar trends again seems to identify this region as sensitive to attentional demands whether due to the presence of a distractor or spatial variability. The absence of an interaction of group for performance sensitivity as well as for brain activity in the presence of significant baseline performance deficits suggests that other factors may contribute to the observed performance deficits in schizophrenia. One possibility is that the right-PFC was unable to recruit connected regions (such as parietal regions and insula) as has been previously proposed^26^ or other regions not accessible to the sensor used in this study. Another possibility is that the vSAT was too easy to elicit a clinically differential response and other modifications may be necessary to capture this aspect. In one recent study investigating the presence of “hyperfocusing” in schizophrenia, Kriether *et al*.^55^ identified that compared to healthy controls, individuals with schizophrenia were unable to properly filter sensory responses to inner vs outer stimuli in a Useful Field of View Task (UFOV). In this work, we observed a tendency towards a performance sensitivity amongst patients between targets which occupied the center position which appeared to support this hypothesis, however, due to the relative rarity of center position targets, the results were inconclusive.

When we examined the interaction of task type and clinical status, we noted significant relations between activity of the left-MFG and clinical status as identified by the Total-PANSS. These relations appeared to be specific to task-related activity associated with the vSAT, with minimal differences observed in the SAT task. When exploring the clinical subscales that contribute to the Total-PANSS score, we saw that this relationship was driven by the PANSS-General and PANSS-Negative subscales with minimal contribution from the PANSS-Positive scale. Several optodes in this region expressed rather strong negative correlations with decreased activity being associated with higher Negative symptom scores. Significant negative correlations in the Left-PFC with PANSS negative symptoms have been previously reported in executive tasks such as the Tower of London^56^ and Trail-Making-Test B^57^. In the vSAT task, this trend was especially strong in Optode 5 for [HbO] (rho = −0.75, p = 0.0001). This region is relatively close to the target of TMS-related therapies^58^ for schizophrenia, which currently is among the only treatment methodologies purporting to ameliorate negative symptoms, although studies report varying efficacy in this matter^58^. Despite this, we observe that mean values in Healthy Controls in this region did not significantly vary from the patient group. These results are more fully explored in the included supporting materials which contains a complete exploration of correlations between behavioral, neurophysiological, and clinical measures (A4).

The clinical symptom correlations of left-PFC region activity and observed differences in right-PFC activity between groups offers supporting evidence of abnormal hemispheric asymmetry in schizophrenia. Early fNIRS studies argued that individuals with schizophrenia had altered asymmetry in executive^59^ and attention tasks^60^, and the reduction of hemispheric connectivity has been repeatedly observed in more recent both structural^61,62^ and functional connectivity^63^ studies. This attenuated asymmetry has been related to increased presence of clinical symptoms^64^ and proposed to be a trait of schizophrenia. Impairments in the right -MFG may more directly influence cognitive control^21,65^ as observed in this task. In patients with severe negative symptoms, failure of activation in the left-MFG may occur as a result of reduced connectivity and reduce the ability of patients to compensate as has been observed when the right-MFG is damaged^66^. Future work should investigate how directed connectivity during cognitive control tasks may influence symptom expression in schizophrenia.

Our study did not show a significant effect for task block number on SAT-Score at any level of stimulus duration in either group (A2). These findings are in support of the work by Demeter *et al*. which concluded in a separate analysis that patients maintained performance across a 12-minute SAT task^22^, showing no appreciable degradation of task performance, whereas children performing the same task were unable to maintain performance. These findings taken together support the existence of general performance deficits in schizophrenia and also provide evidence that attentional differences in schizophrenia are more closely related to difficulties adapting to varying demands in attentional processing rather than issues associated with fatigue^10,67^.

Findings in the study while promising are subject to several limitations. First, all patients in the study were medicated and it is possible that the effects of varied prescribed antipsychotics may have contributed to the results observed in the study. Although preliminary analysis indicated that patient performance was not sensitive CPZ equivalent dosage, antipsychotic medication has commonly been reported to have limited effect on cognitive measures including cognitive control and attention^68,69^. Second, the block -level analysis method used in this study may have overlooked interesting features which could have provided more information regarding visuospatial deficiencies in schizophrenia. Future researchers may wish to use systems capable of higher sampling frequencies to investigate event-related responses within trials of the SAT and vSAT. It has been proposed that higher sampling frequencies may increase the speed of fNIRS response detection^70^ and other more rapid imaging techniques such as electroencephalography (EEG) may offer additional insight into the visuospatial dysfunction observed here. Third, the fNIRS system used in the study did not allow regions outside the anterior prefrontal cortex to be monitored. It is possible that other brain areas such as parietal cortex might provide additional information. Lastly, schizophrenia is also accompanied by visual processing deficits in a variety of modalities which contribute to higher level dysfunctions in a variety of downstream domains^71,72^. Performance differences frequently attributed to failures of attention and other top-down processes may be conflated with dysfunction in the visual processing system^73^. The magnocellular pathway is critical for the rapid detection of low-contrast and stimuli with low spatial frequency. When working together with the slower parvocellular pathway, the two integrate to form a complete visual representation. In this “fill and frame model” visual, the magnocellular pathway identifies broad visual descriptions while the parvocellular pathways provide the details. Patients with schizophrenia possess a demonstrated degradation in the magnocellular pathway that results in reduced visually evoked responses in situations with low contrast (<10% luminance), short stimulus length, and low spatial frequency^73,74^.

In the presently reported experiment, the varied stimuli locations in the vSAT task when paired with reduced contrast stimuli may have contributed to performance differences between patients and healthy controls. However, we suggest that differences due to magnocellular deficits may only be apparent at the shortest interstimulus intervals. The experimental setup used (single monitor at a comfortable distance from subject) in this study was not designed in a manner which would have field of view to be sensitive to the difference between magnocellular and parvocellular processes. Although we note that performing this experiment using a display arrangement that is capable of targeting peripheral vision (>30 deg) may be an interesting avenue of research. A combination of attentional tasks and manipulation of low-level visual-detection could provide a novel evaluative technique pairing low and high-level brain function.

## Conclusion

In summary, we confirmed a general attentional deficit in patients with schizophrenia as demonstrated by decreased task performance and increased response time compared with the healthy controls. In both healthy and patient populations, we found a decrease in performance associated with task-type manipulation and a change in the target stimulus duration, but task parameters did not interact with subject group. The results confirm that the right frontopolar region plays a role in control of attention for both patients and healthy controls. However, patients with schizophrenia exhibited a general attentional deficit and inefficient right-medial PFC activation. Finally, we observed a strong regional association between the left-MFG activity during the vSAT task and the PANSS score driven by the negative symptom subscale. We suggest that the presence of aberrant activation differences within the left-MFG region may be related to dysregulation of attentional networks which plays a role in the clinical expression of negative and general symptoms.

## Supplementary information

Appendix

## Data Availability

The datasets generated during and/or analyzed during the current study are available from the corresponding author on reasonable request.
