# Supplementary material for: Visuospatial task-related prefrontal activity is correlated with negative symptoms in schizophrenia
Source: Sci Rep. 2019 Jul 3;9:9575. doi: 10.1038/s41598-019-45893-7 (PMC6610077; doi:10.1038/s41598-019-45893-7)
Supplement: Supplementary file 1 — Appendix [file 41598_2019_45893_MOESM1_ESM.docx]

Supplementary Information

for

**Visuospatial task-related prefrontal activity is correlated with negative symptoms in Schizophrenia**

Adrian Curtin^1,2^, Junfeng Sun^2^, Qiangfeng Zhao^2^, Banu Onaral^1^, Jijun Wang^3^*, Shanbao Tong^2^*_,_ Hasan Ayaz^1,4,5^*

1 Drexel University, School of Biomedical Engineering, Science and Health Systems, Philadelphia, PA, USA

2 Shanghai Jiao Tong University, School of Biomedical Engineering, Shanghai, China

3 Shanghai Mental Health Center, Shanghai Jiao Tong University, School of Medicine, Shanghai, China

4 University of Pennsylvania, Department of Family and Community Health, Philadelphia, PA, USA

5 Children’s Hospital of Philadelphia, Center for Injury Research and Prevention, Philadelphia, PA, USA

Contents

[A1. Behavioral Performance in terms of SAT-score and d-prime 2](#_Toc10483703)

[A2. Behavioral Performance over session 4](#_Toc10483704)

[Task Performance between sessions was not influenced by block number or signal duration 4](#_Toc10483705)

[Response Time demonstrated clear improvements with practice despite stable performance 4](#_Toc10483706)

[A3. Comparative Literature Results 5](#_Toc10483707)

[Healthy Controls performed similarly to prior literature on SAT tasks 5](#_Toc10483708)

[Patients showed reduced performance, but similar sensitivity to vSAT and dSAT conditions 6](#_Toc10483709)

[A4. Clinical and Behavioral Correlational Analyses 7](#_Toc10483710)

[Minor Correlations within Clinical Terms and between Behavioral and Clinical Terms 7](#_Toc10483711)

[Cortical Response Correlates with Behavioral Performance Measures 7](#_Toc10483712)

[vSAT Activity Expresses Strong Correlations with PANSS Negative Subscale 7](#_Toc10483713)

[Cortical correlations observed with Duration of Illness and Antipsychotic dosage 8](#_Toc10483714)

[A5. Representative fNIRS Time-series 8](#_Toc10483715)

[A6. Detailed Pharmaceutical Dosage of Clinical Group 8](#_Toc10483716)

[References 9](#_Toc10483717)

## A1. Behavioral Performance in terms of SAT-score and d-prime

Although the SAT-Score is a useful indicator of task performance and vigilance, simple classification accuracy can be a more interpretable measure of performance during the task. This metric produced similar trends in between groups and durations as shown by SAT-Score. These trends can be examined in Figure A1.

Accuracy was calculated as the number of correct hits and correct rejections over the total number of stimulus trials.


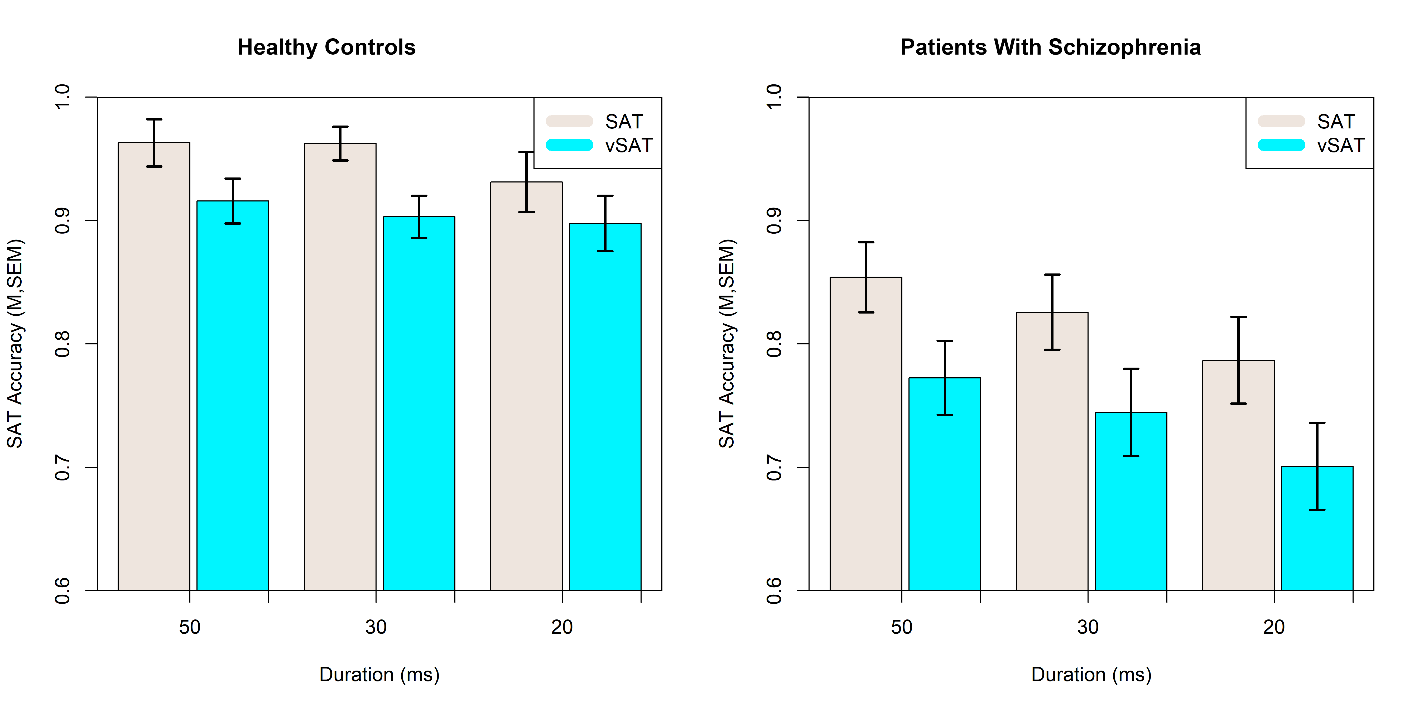


Figure A1: Effects of variable task load and stimulus duration on the Accuracy by healthy adult controls and patients with schizophrenia

The sensitivity index (d’ or d-prime) forms a statistic typically used in signal detection theory describing the separation of the statistical distribution of the noise and signal means. D-prime is typically estimated as

$$\boldsymbol{d}^{\boldsymbol{'}}\boldsymbol{=Z}\left( \boldsymbol{Hits} \right)\boldsymbol{-Z(False Alarms)}$$

Where the function Z is the inverse of the cumulative distribution function of the Gaussian distribution. d-prime is a nonparametric measurement describing how detectable a signal amongst the noise with higher values indicating a more observable signal.


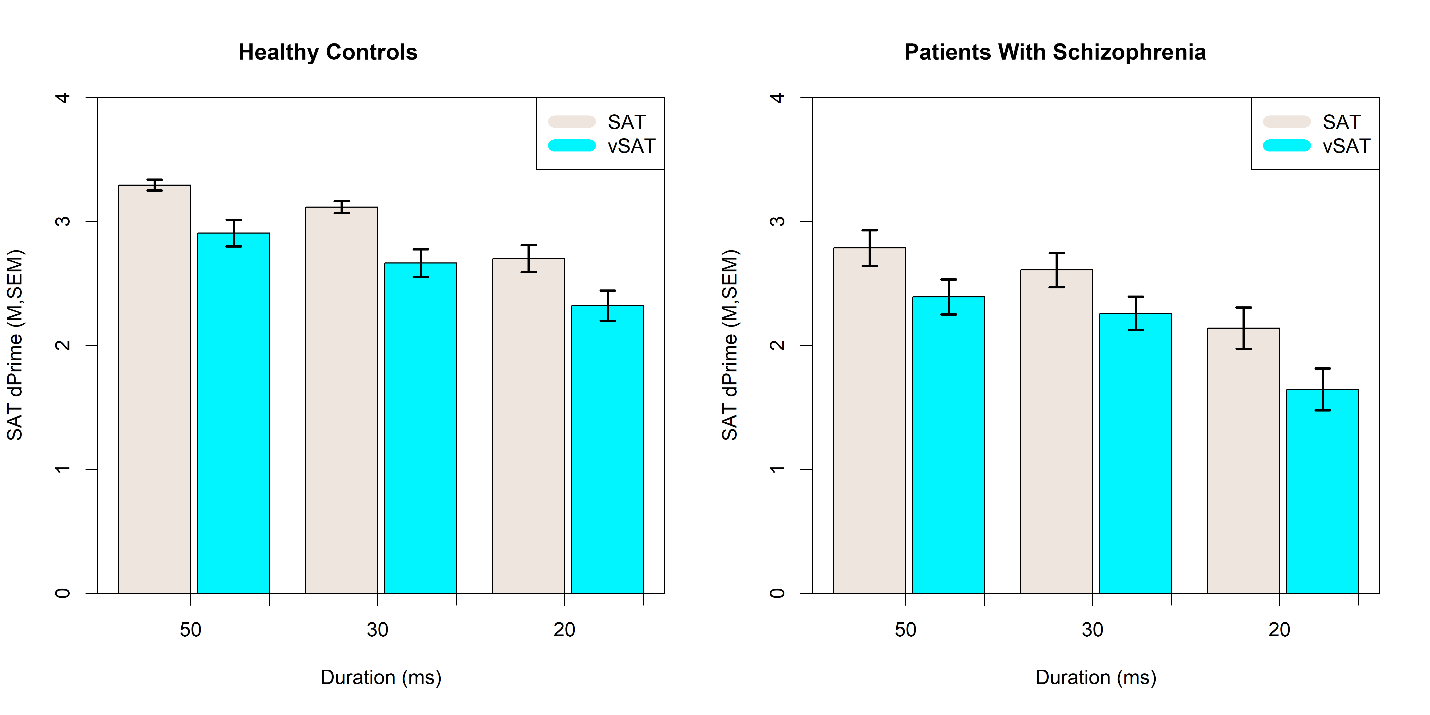


Figure A2: Effects of variable task load and stimulus duration on the sensitivity index (d-prime) by healthy adult controls and patients with schizophrenia

Both methods identified similar term selection in the LME model analysis with significant effects for Group (p~=0.0029~0035), Task-Type (p<0.0001), and Duration (p<0.0002), but retaining no interaction terms in the minimal model.

Table A1: LME model terms for Alternate Performance Metrics

| Outcome | |  | Main Effects | | | |  | Parameter Estimates | | | |  |
| --- | --- | --- | --- | --- | --- | --- | --- | --- | --- | --- | --- | --- |
|  |  |  | df_  num | df_  den | F | p |  | Linear |  | Quadratic |  |  |
| *Accuracy* | |  |  |  |  |  |  |  |  |  |  |  |
|  | Intercept |  |  |  |  | 0.00000 | *** | 0.9308 | (0.0382) |  |  |  |
|  | Group |  | 1 | 37.12 | 10.224 | 0.00283 | ** | -0.1580 | (0.0494) |  |  |  |
|  | Task-Type |  | 1 | 393.5 | 33.46 | 0.00000 | *** | -0.0483 | (0.0084) |  |  |  |
|  | Duration |  | 2 | 389.2 | 8.832 | 0.00018 | *** | -0.0363 | (0.0087) | 0.0058 | (0.0087) |  |
| *dPrime* | |  |  |  |  |  |  |  |  |  |  |  |
|  | Intercept |  |  |  |  | 0.00000 | *** | 2.7861 | (0.1686) |  |  |  |
|  | Group |  | 1 | 37.22 | 9.723 | 0.00350 | ** | -0.6796 | (0.2180) |  |  |  |
|  | Task-Type |  | 1 | 263.96 | 44.381 | 0.00000 | *** | -0.2915 | (0.0438) |  |  |  |
|  | Duration |  | 2 | 389.06 | 41.277 | 0.00018 | *** | -0.4120 | (0.0467) | -0.0997 | (0.0467) |  |
| Denominator Degrees of freedom calculated with Satterthwaite approximation | | | | | | | | | | | | |
| Random effects specified as random intercept and slope for each participant | | | | | | | | | | | | |
| † p<0.1 | ** p<0.01 | | | | | | | | | | | |
| * p<0.05 | *** p<0.001 | | | | | | | | | | | |

## A2. Behavioral Performance over session

### Task Performance between sessions was not influenced by block number or signal duration

As noted in the main body, inclusion of block number in the LME model did not provide either a significant fixed effect for block number (F(1,37.09) = 0.079, p>0.7) or an improved model (ChiSq = 0.837, p>0.7) suggesting that SAT-Score was not influenced by block number. This perspective is presented in Figure A3.


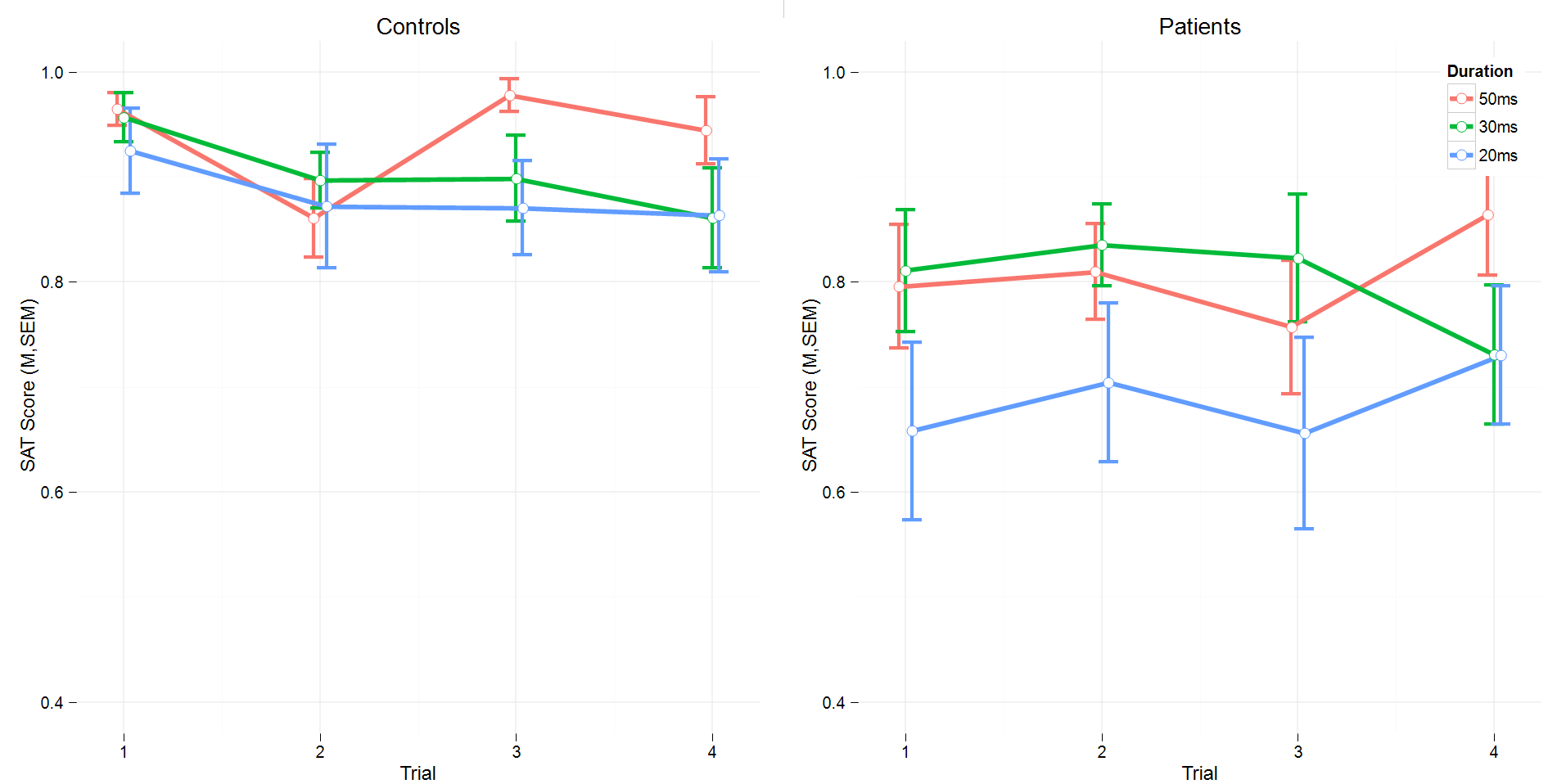


Figure A3: SAT scores suggest minimal influence by task block number during task performance when averaged across task blocks

### Response Time demonstrated clear improvements with practice despite stable performance

Unlike score, block number had a clear influence on Response Time as is visible in Figure A4. Since Block was used as a random effect in the LME model, these changes can be considered as adjusted for in the statistical model. Clear differences between Subject groups can be observed when examining between each block number. It is noted that as the SAT is an unhurried task, improvement of the speed of response did not necessarily translate into improved classification accuracy or score.


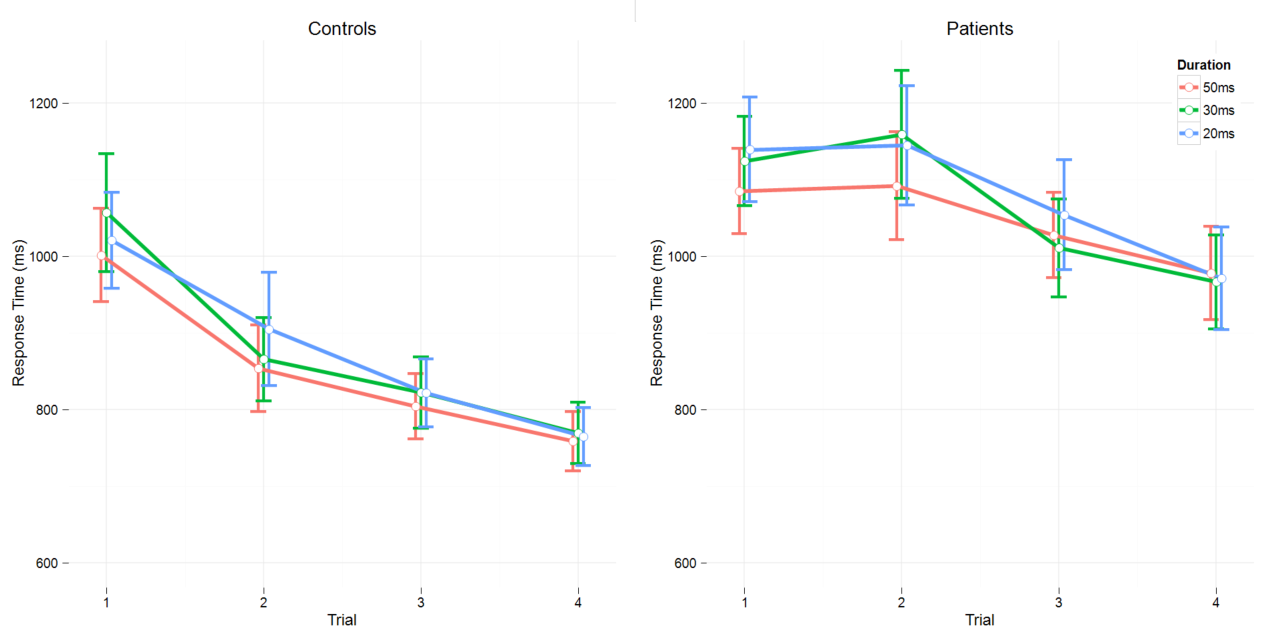


Figure A4: SAT Response Times Suggest that both Healthy and Patient subjects improved task performance in terms of reaction speed, however Healthy subjects demonstrated larger improvements

## A3. Comparative Literature Results

### Healthy Controls performed similarly to prior literature on SAT tasks

The distracted-SAT (dSAT) is a task condition meant to interfere with “control of attention” through the addition of a 10Hz flashing background over which the signal is presented. By introducing a salient distraction to the monitoring task, an additional burden is placed on the individual’s ability to adequately control their attentional aspects. Recent research has reported that this distraction modulation separates behavioral healthy control performance from patients with schizophrenia^1^. In order to validate our implementation of the SAT task and the vSAT task, our results are compared with the previously published work by Demeter et al. ^1^ which included the SAT task, distracted SAT (dSAT) and variable spatial location SAT (vsl-SAT). These behavioral measures were reported in an appendix in the form of “Hits-To-Target”, a measure used in the calculation of SAT-score, and were compared using unpaired t-tests for each stimulus duration.


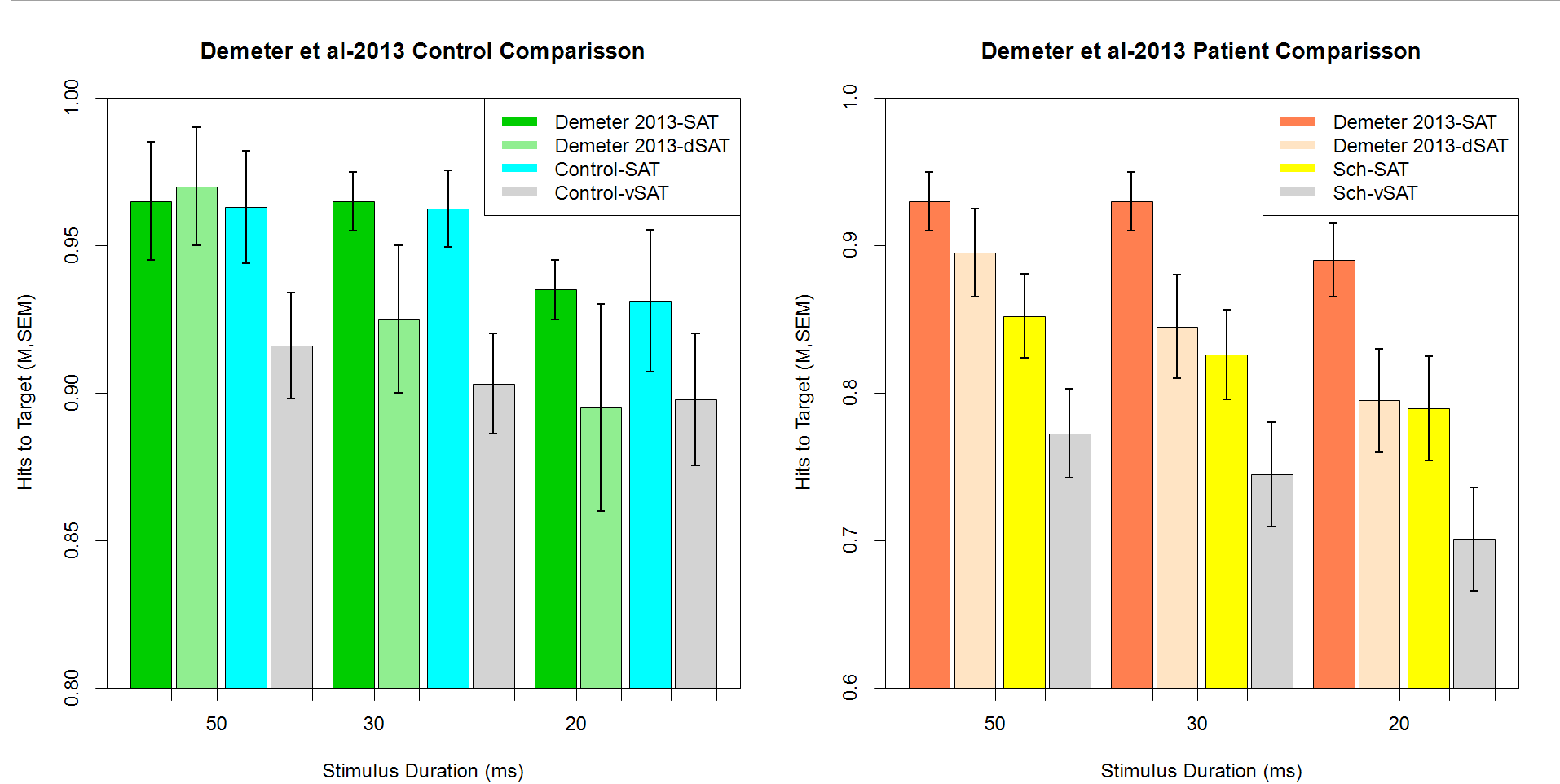


Figure A5: Comparative Literature Analysis with behavioral performance reported in Demeter et al^1^. A) Comparisons for healthy controls between studies, B) Comparisons between patients with schizophrenia.

For Healthy Subjects, the presently reported control group performed almost identically in the SAT-task for all stimulus durations (p>0.85), and while “Hits-To-Target” for the vSAT task was numerically lower than the corresponding VSL group for all durations, these differences did not reach significance (p>0.15). Additionally, performance of the vSAT task was similar to performance during the distractor condition for the non-VSL (p=0.42) group and for the VSL (p=0.39) for all durations except the 50ms duration in which the vSAT-task condition was significantly lower than the corresponding dSAT-task condition (p<0.01). These results suggest that the SAT task was implemented in a similar manner to the prior study, and that for healthy subjects, the introduction of the vSAT task type produced similar performance reductions as those introduced by either the VSL condition or the distractor condition SAT. Comparison of control group performance is presented in Figure A5a.

### Patients showed reduced performance, but similar sensitivity to vSAT and dSAT conditions

Comparisons between the patient groups in both studies revealed that the presently-reported patient group performed significantly worse than patients who participated in the prior study during the SAT task condition (p<0.05). In comparisons between the dSAT and vSAT performance, significant performance differences were observed for the 50ms stimulus duration (p<0.01) and while performance between conditions approached significance for the 30ms (p=0.052) and 20ms (p=0.068) conditions respectively. When comparing task performance reductions as a result of condition change (vSAT and dSAT respectively), performance reductions associated with the dSAT task condition were most similar for the 30ms (p>=0.97) and 20ms (p>=0.89) durations whereas the 50ms was not as similar (p=0.27) because patient’s performance in the dSAT condition did not differ significantly from the SAT condition at the 50ms interval. This similarity was also observed in the control group of the same study. From these findings, we observe that while overall performance in-between the studies was reduced for the patient group, the performance reductions associated with modulating task-conditions (vSAT and dSAT) were similar. Results are presented in Figure A5b.

It was proposed that, because bottom-up influence would contribute a constant effect on task performance, the SAT can be seen as a general measure of attentional performance and the vulnerability to task condition could be used as a unique indication of input selection problems. However, currently it is not clear whether the introduction of the distractor would simply increase attentional demands by placing a separate burden from the original attention task.

## A4. Clinical and Behavioral Correlational Analyses

In order to fully explore the relationship between task performance and the contributions of clinical factors on cortical responses, we assessed the correlation between individual variables and their associated biomarkers. In patients we examined relationships between Score and Response Time, PANSS total score, as well as the PANSS general, negative and positive subscales. We also investigated relationships with duration of illness and antipsychotic dosage as reported in Chlorpromazine (CPZ) equivalent units. Relationships for explored correlated variables were examined for both Task Types individually, as well as across the mean for both Tasks.

### Minor Correlations within Clinical Terms and between Behavioral and Clinical Terms

Within the clinical data we observed that PANSS negative correlated with PANSS general (rho=0.46, p=0.024) and PANSS positive approached a significant correlation with PANSS general (rho=0.399, p=0.0535). However, Duration of Illness and CPZ-equivalent dosage did not correlate significantly with PANSS or each other. The behavioral metrics of Score and Response Time, did not correlate significantly with clinical scales, Duration of Illness or CPZ-equivalent dosage in either task, although PANSS general approached significant correlation with mean Response Time (rho=0.36, p=0.086). ScoreDifference (SAT-vSAT) also approached significance for PANSS positive (rho=-0.39, p=0.060).

### Cortical Response Correlates with Behavioral Performance Measures

SAT Score in the vSAT task showed a significant negative correlation with Optode 15 for [HbO] (rho=-0.54, p=0.03). Score in the SAT task showed significant correlations with Optodes 2 and 16 (rho=0.493~51, p=0.0269~0.0313). Mean Response Time during the vSAT condition correlated significantly with Optode 7 for [HbO] (rho=-0.5, p=0.049), Optode 4 for [Oxy] (rho=-0.47, p=0.41), and Optode 9 for [HbR] (rho=-0.51, p=0.287. During the SAT condition mean RT correlated significantly for [HbR] with Optodes 13 (rho=-0.56, p=0.025) and 16 (rho=-0.67, p=0.0013).

### vSAT Activity Expresses Strong Correlations with PANSS Negative Subscale

Total PANSS correlated significantly with Optode 1 for [Oxy] on both SAT and vSAT conditions (rho=-0.57~0.6, p=0.042) but Optode 1 was missing many samples due to automated quality rejection. Optodes 5 and 6 correlated significantly with Total-PANSS score on [HbO] (rho=-0.54~-0.58, p=0.014~0.02) and Optode 14 for [HbR] (rho=-0.55, p=0.03) for vSAT and Optode 16 for [HbR] (rho=-0.47, p=0.04) for the SAT condition.

The PANSS Positive subscale correlated with elicited activity from the SAT task on Optodes 1,3, and 5 for [HbO] (rho=0.45~0.6, p=0.028~0.041), and Optode 9 for [HbR] (rho=0.49, p=0.040). The PANSS General subscale correlated with Optode 1 for [Oxy] (rho=-0.6, p=0.039) and Optodes 6, 7, and 8 for [HbO] (rho=-0.46~0.55, p=0.018~0.046) for the vSAT condition.

For the PANSS negative subscale, no significant relationships were observed during the SAT task, but correlations in the vSAT were much more prominent. PANSS-negative correlated negatively with [HbO] for Optodes 5, 6, 7, and 9 (rho=-0.522~-0.776, p=0.0002~0.0377). [Oxy] also exhibited strong negative correlations with PANSS negative in Optodes 5 and 6 (rho=-0.58~-0.65, p=-0.004~0.011). Parametric visualizations of [HbO] correlations with PANSS subscales and selected correlations from Optode 5 are visualized in Figure A6. Despite these strong correlations, particularly in Optode 5, there were no significant differences between mean evoked responses between groups identified in the linear models.

### Cortical correlations observed with Duration of Illness and Antipsychotic dosage

Duration of Illness correlated with evoked activity for Optode 16 for [HbO] (rho=-0.59, p=0.0056), Optode 7 for [HbR] (rho=-0.54, p=0.021) and Optode 12 for [Oxy] (rho=-0.518, p=0.0277) during the SAT task. During the vSAT task, only Optode 16 for [Oxy] correlated significantly with Duration Ill (rho=0.46, p=0.29).

CPZ-equivalent dose showed significant correlations for Optode 4 for [HbO] (rho=0.46, p=0.048) and [Oxy] (rho=0.51, p=0.025), as well as Optode 15 for [Oxy] (rho=0.52, p=0.038), and Optodes 8 (rho=-0.55, p=0.018) and 10 (rho=-0.55, p=0.021) for [HbR] during the vSAT. During the SAT task, dosage correlated significantly for Optodes 1 (rho=0.58~0.6, p=0.031~0.36) and 10 (rho=0.5~0.61, p=0.011~0.048) for both [HbO] and [Oxy] biomarkers.

## A5. Representative fNIRS Time-series

Figure A6. A presentative hemodynamic time-series ([HbO], [HbR] and [Oxy]) during the SAT task.

## A6. Detailed Pharmaceutical Dosage of Clinical Group

Table A2. Drug and dosage information for patients with schizophrenia

|  | **Pharmaceutical Information** | | |
| --- | --- | --- | --- |
| **Patient** | **Drug** | **Dose (mg/day)** | **Equiv (100mg CPZ)** |
| 1 | quetiapine | 400 | 200 |
| 2 | clozapine | 400 | 800 |
| 3 | risperidone | 4 | 200 |
| 4 | clozapine | 75 | 150 |
| 5 | paliperidone | 9 | 600 |
| 6 | risperidone | 4 | 200 |
| 7 | risperidone | 4 | 200 |
| 8 | paliperidone | 9 | 600 |
| 9 | aripiprazole | 10 | 133 |
| 10 | risperidone | 3 | 150 |
| 11 | risperidone | 3 | 150 |
| 12 | olanzapine | 7.5 | 150 |
| 13 | olanzapine | 20 | 400 |
| 14 | risperidone | 4 | 200 |
| 15 | aripiprazole | 30 | 400 |
| 16 | risperidone | 4 | 200 |
| 17 | olanzapine | 10 | 200 |
| 18 | risperidone | 4 | 200 |
| 19 | olanzapine | 25 | 500 |
| 20 | paliperidone | 6 | 400 |
| 21 | clozapine | 100 | 200 |
| 22 | risperidone | 6 | 300 |
| 23 | olanzapine | 20 | 400 |
| 24 | risperidone | 4 | 200 |

# References

1. Demeter, E., Guthrie, S. K., Taylor, S. F., Sarter, M. & Lustig, C. Increased distractor vulnerability but preserved vigilance in patients with schizophrenia: Evidence from a translational Sustained Attention Task. *Schizophr. Res.* **144,** 136–41 (2013).
